# Pediatric Multiple Sclerosis in Tunisia: A Retrospective Study over 11 Years

**DOI:** 10.1155/2017/4354826

**Published:** 2017-11-07

**Authors:** Nedia Ben Achour, Ibtihel Rebai, Sarra Raddadi, Hanene Benrhouma, Hedia Klaa, Aida Rouissi, Ichraf Kraoua, Ilhem Ben Youssef Turki

**Affiliations:** ^1^Research Unit UR12 SP24 and Department of Child and Adolescent Neurology, National Institute Mongi Ben Hmida of Neurology, Tunis, Tunisia; ^2^Faculty of Medicine of Tunis, Tunis El Manar University, 1007 Tunis, Tunisia

## Abstract

**Introduction:**

Pediatric multiple sclerosis (pMS) is a rare demyelinating disorder with an onset before the age of 18 years. In this study, we aimed to investigate the characteristics of pMS in Tunisian children.

**Patients and Methods:**

We conducted a retrospective study over 11 years (2005–2016) including all patients diagnosed with pMS according to the International Pediatric Multiple Sclerosis Study Group (IPMSSG) criteria of 2012 and followed up in a tertiary care research center. Epidemiological, clinical, neuroimaging, laboratory, and therapeutic data were collected and analyzed.

**Results:**

There were 21 patients. The male-female ratio was 1 : 3. Mean age at onset was 11 years (range: 3–17 years). Three patients had type 1 diabetes. Polyfocal presentation was preponderant (81%) with motor dysfunction in 57% of patients. Paroxysmal dystonia was noticed in 24%. All patients were diagnosed with relapsing-remitting form. Interferon beta was prescribed in 80% with a reduction of annual relapse rate.

**Conclusion:**

The annual incidence of pMS in Tunisian children aged below 18 years could be estimated as 0.05 per 100,000. Singular features in our cohort were the frequent association with type 1 diabetes and the increased occurrence of dystonia. Greater awareness of pMS may be helpful to improve management strategies of children and their families.

## 1. Introduction

Pediatric multiple sclerosis (pMS) is a rare demyelinating disorder of the central nervous system defined by an onset before the age of 18 years. Diagnosis was based historically on the adult's criteria and children were treated using adult paradigms. It is recognised as multifactorial disease, caused by genetic vulnerability and environmental triggers that are still investigated [1]. In Tunisia, there are no accurate pMS epidemiological data. In this study, we aimed to investigate demographic characteristics, clinical features, biological and radiological aspects, management, and outcome of Tunisian children with pMS.

## 2. Patients and Methods

### 2.1. Setting

We conducted a retrospective and descriptive study over 11 years (between 1 January 2005 and 31 December 2016) in the Department of Child and Adolescent Neurology at the National Institute Mongi Ben Hmida of Neurology (Tunis, Tunisia). The total population of our country (2005–2016) is 10618933 ± 399773, with 95% confidence interval (CI) of 10.618.863–10.619.002. The pediatric population aged below 18 years (2005–2016) accounts for 3.490.333 ± 62128, with 95% CI of 3.490.314–3.490.352. Our department is a referral tertiary care and research center for children and adolescents with neurological disorders from the whole country and serves a population of over three million people (aged below 18 years). More detailed information about demographics of Tunisia and presentation of our center is provided in Supplementary Material available online at https://doi.org/10.1155/2017/4354826.

### 2.2. Inclusion Criteria

All patients managed in our department for acquired demyelinating syndromes (ADS) have been retrospectively reviewed. We included in our study all patients who fulfilled the International Pediatric Multiple Sclerosis Study Group's (IPMSSG) revised criteria (2012) [2] for pMS with a minimum follow-up period > 6 months.

### 2.3. Exclusion Criteria

All patients with genetic leukoencephalopathies and demyelinating disorders such as leukodystrophies, aminoacidurias, and mitochondriopathies have been excluded. Patients who presented with acquired demyelinating disorders mimicking MS and whose final diagnoses were infectious, vascular, nutritional (B12 or folate deficiency), or inflammatory disorders (systemic lupus erythematosus, neurosarcoidosis, neuro-Behçet, Sjogren's syndrome,…) have been excluded based on neuroimaging findings and biological and immunological tests. We excluded also all patients with other ADS (CIS, NMO, ADEM,…) who did not fulfill the MS criteria.

### 2.4. Procedures

Medical records of patients with pMS were retrospectively reviewed. Demographic characteristics, clinical data, Expanded Disability Status Scale (EDSS) score at first consultation and at the last one, biological findings, characteristics of the first magnetic resonance imaging (MRI), and the data about therapeutic management and outcome were collected.

A descriptive analysis was performed using SPSS software.

The study was conducted in accordance with the principles of the Declaration of Helsinki.

## 3. Results

Among 47 patients with ADS, 21 patients fulfilled the diagnosis criteria for pMS and were included in our study. The male-female ratio was 1 : 3. Sixteen patients (76%) originated from northern regions, three from the center (14%), and two from southern regions (10%).

Mean age at onset was 11 years (range: 3–17 years). Time between the first symptom and the diagnosis of MS varied between 1 day and 2 years.

Medical history corresponded to type 1 diabetes in three cases (10%) and hypothyroidism, factor 7 deficiency, allergic rhinitis, neonatal jaundice, and hyperpyretic seizures in one case, respectively (4%).

First onset symptoms occurred in winter and in summer in six patients (28%), respectively, in autumn in five (23%) cases, and in spring in four cases (19%). The mean follow-up period was three years (ranging from 6 months to 8 years).

Polyfocal presentation was noticed in 17 patients (81%), whereas monofocal presentation was present in 4 patients (19%). The most preponderant clinical manifestations were motor dysfunction in 12 patients (57%), ataxia and brainstem symptoms in 10 patients (48%), respectively, and sensory dysfunction in 8 patients (38%). Optic neuritis was documented on visual evoked potentials in 13 patients (62%). Five patients (24%) had movement disorders as paroxysmal dystonia. An acute demyelinating encephalomyelitis- (ADEM-) like presentation was noticed in one patient (5%).

Demographic and clinical data were presented in Table 1.

**Figure 1:**
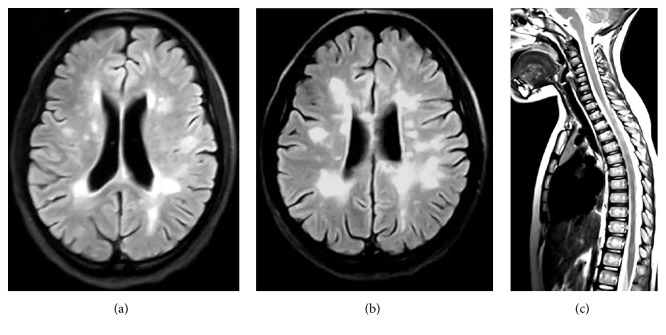
Axial FLAIR weighted images ((a) and (b)) showing multiple hyperintense lesions on periventricular and subcortical regions in patient 5 with spinal dorsal involvement on sagittal T2 weighted image (c).

On their first MRI, all patients showed increased signal on FLAIR and T2 weighted images (Figure 1) with supratentorial lesions in all cases (100%) and infratentorial involvement in 17 cases (81%). Black holes corresponding to hypointense lesions on T1 weighted images were present in all cases reflecting the axonal injury. Spinal involvement was noticed in 11 cases (52%) with cervical location in 6 cases (29%). Eighteen patients (86%) had gadolinium enhancement on T1 weighted images revealing active inflammatory lesions.

CSF analysis was performed in 20 patients (95%). On their first lumbar puncture, only 8 patients (38%) showed positive oligoclonal bands (OCB). An increased CSF cell count (less than 30 lymphocytes/mm^3^) has been noticed in 5 cases (25%). Patients with negative OCB and/or increased cell count had a second lumbar puncture which revealed positive OCB in 85%.

Detailed neuroimaging and laboratory data were presented in Table 2.

In order to exclude differential diagnosis of MS, all our patients (especially those with increased CSF protein and cell count) had serum and CSF screening for HSV, EBV, VZV, CMV, HIV, rubella virus, hepatitis B and C, mycoplasma, West Nile, HIV, Lyme, and syphilis and showed no argument for recent infection.

Immunological tests (antinuclear, antirheumatoid factor and anti-Sm antibodies) and angiotensin-converting enzyme showed normal results in all patients.

Patients with movement disorders had a screening for Wilson's disease (serum ceruloplasmin test; serum and urine copper tests) and mitochondriopathy (lactate in serum and CSF) and some patients had screening for NMDA antibodies and streptolysin O antibodies.

Children with a very early onset (patients 3, 8, 15, and 19) had assessment of ammonia, lactate (serum and CSF), amino acid, and urine organic acid chromatography. All these biological investigations showed normal results.

Other biological investigations were performed and aimed to evaluate risk factors for MS. They mainly included measurement of serum 25-hydroxyvitamin D3 (25(OH)D) levels performed in 9 patients. The levels indicated a deficiency state (defined as 25(OH)D levels less than 10 ng/ml) in 6 patients with a lowest rate of 6 ng/ml. Vitamin D insufficiency (defined as 25(OH)D levels between 10 and 20 ng/ml) was found in one patient (18.8 ng/ml).

All patients had been diagnosed with relapsing-remitting MS (RRMS).

Seventeen patients enrolled in the study underwent immunomodulatory therapy with interferon *β* 1a in 15 cases and interferon *β* 1b in two cases. The EDSS score was 3 at the onset (range: 1.5–6) and 1 at the last evaluation (range: 0–3.5). The median annual relapse rate before and after immunomodulatory therapy was, respectively, 2 (range: 0.25–4) versus 0.42 (range: 0–1.33). Only one patient developed a secondary progressive form.

Therapeutic and follow-up data were presented in Table 3.

## 4. Discussion

Our study provides demographic, clinical, biological, and radiological characteristics, management, and outcome of pMS in Tunisia over 11 years.

Annual incidence of pMS per 100,000 children varies across studies and countries. It has been estimated to be 0.13 in France, 0.18 in Canada, and 0.51 in USA and increased to 2.85 in Sardinia (Italy) [1, 3, 4]. In Tunisia, there are no accurate pMS epidemiological data. Given the demographic characteristics in our country, the annual incidence of pMS in Tunisian children aged below 18 years could be estimated as 0.05 per 100,000. In patients aged below 15 years, it could be estimated as 0.04 per 100,000 and it increased to 0.08 per 100,000 when considering patients aged between 15 and 18 years. These findings highlight the rarity of pMS in our country [1, 3, 4] supporting the north-south gradient of MS incidence.

The median age of onset of pMS symptoms in other reported studies ranges between 9.3 and 15.5 years (11 years in our patients) and the median age at diagnosis is about 16 years [1]. A very early onset as found in four of our patients (patients 3, 8, 15, and 19) has been reported [5]. Meanwhile, a very early onset calls to mind the possibility of inherited leukoencephalopathies or inborn neurometabolic disorders and practitioners should be very careful and cautious before retaining the diagnosis of MS.

As shown in our study, pMS is 1.5–2 times more common in female than in male children, suggesting the hormonal pubertal influence on the onset of the disease, whereas the sex ratio is 1 when the disease begins before the age of 10 [6].

A frequent association with type 1 diabetes was found in 14% of our patients. Both MS and type 1 diabetes are considered as autoimmune disorders with different clinical manifestations. Although pathophysiological mechanisms underlying this association are still unclear, some studies suggest the implication of HLA system particularly the HLA-DRB1*∗*1501 allele and the deficiency of vitamin D [7, 8].

According to previous reports, 50 to 70% of patients have a polyfocal presentation as in our series with preponderance of motor dysfunction. Monofocal presentations as optic neuritis or transverse myelitis are less frequent in pMS (around 10%). ADEM-like presentation is more likely to occur in younger patients displaying polyfocal symptoms with altered behavior or consciousness, which was observed in one patient of our series.

The high occurrence of paroxysmal dystonia (24%) seems to be a characteristic feature in our patients, whereas it was reported in only 2.9% of adult MS series and has unknown frequency in the pediatric population [9]. The underlying mechanisms may be related to contiguity of demyelinating lesions to basal ganglia pathways. Interestingly, dystonia was present mainly at onset and was responsive to corticosteroids ± carbamazepine.

Neuroimaging features of pMS are quite similar to those in adult MS including multiple demyelinating lesions in the periventricular regions, the corpus callosum, and the spinal cord. However, brain lesions in younger children (age < 11 years) may be large and confluent with poorly defined borders at the onset of disease mimicking ADEM lesions. Infratentorial involvement noticed in 81% of our patients seems characteristic in pMS patients, suggesting the preferential immune targeting of mature myelin [1, 10].

CSF analysis, which provides information about the inflammation process, reveals normal cell count and protein level in almost 60% of cases. Increased CSF cell count less than 30 lymphocytes/mm^3^ may be noticed in some children with MS as reported by Sudhakar et al. [10] and as found in our patients. A higher CSF cell count is more suggestive of other diagnoses and requires exclusion of central nervous system infection, vasculitis, autoimmune systemic disorders, or neuromyelitis optica (NMO) [11]. Although OCB are not specific for inflammatory demyelination, they are useful in supporting diagnosis. Sensitivity on initial lumbar puncture varies between 35% (young children) and 68% (adolescent), which is not as high as in adult MS (90%). As reported in our study, some pMS patients who were OCB-negative at the onset become OCB-positive during follow-up [1, 3, 10].

To the best of our knowledge, there are no randomized controlled studies regarding the disease modifying therapies in pMS which intend to prevent relapses. Thus, immunomodulatory therapies are derived from adult clinical trials and small retrospective, observational studies.

Interferons or glatiramer acetate is considered as the first-line therapy with proven efficacy and safety [1]. In our series, 81% of our patients underwent immunomodulatory treatment based on interferon beta with no side effects reported in all of them.

A RRMS form was diagnosed in all patients of our series, which is in agreement with literature, where RRMS form is higher than 80% in the pediatric population [3, 12, 13]. After the use of modifying therapy, only one patient developed a secondary progressive form.

Consistently with previous reports, a reduction in relapse rate after the initiation of first-line treatments was noticed passing from 2 relapses per year to less than 1 relapse per year. However, some children may present with refractory disease or have poor tolerance and switch to another first- or a second-line therapy as natalizumab, daclizumab, cyclophosphamide, or fingolimod but with an increased risk of side effects when the latter are used which should be balanced against potential benefits. Recent studies report that annual relapse rate seems significantly higher in pMS in comparison with adult MS (1.13 versus 0.4), which may be due to the presence of more central nervous system immune cells in the pediatric population with more intense inflammatory reaction, particularly in young children [14].

## 5. Conclusion

Our study illustrates pMS characteristics in Tunisia. The annual incidence of pMS in Tunisian children aged below 18 years could be estimated as 0.05 per 100,000. Our results are similar to previously reported studies regarding clinical, neuroradiological, and laboratory features which support the different characteristics of pMS compared to the adult form. However, singularity in our cohort was the frequent association with type 1 diabetes as a risk factor for MS and the increased occurrence of dystonia. The underlying mechanisms of pMS are yet to be elucidated requiring further immunobiological research. Thus, greater awareness of this demyelinating disorder may be helpful to better understand the underlying mechanisms and to improve therapeutic and management strategies of children and their families.

## Supplementary Material

More detailed information about demographics of Tunisia and presentation of our center.

## Figures and Tables

**Table 1:** Demographic and clinical findings in our series.

	Gender	Age of onset (years)	Family history	Medical history	Inaugural symptoms	Clinical manifestations on first attack, EDSS score
Patient 1	F	17	Goitre	None	Dysarthria + weakness of lower limbs + numbness in upper limbs	Thermic hyperesthesia + tactile hypoesthesia + upper limb dystonia + cerebellar ataxia. EDSS = 3.5

Patient 2	F	16	Epilepsy	Allergic rhinitis	Facial paralysis + diplopia + dizziness	6th nerve paralysis + tetrapyramidal syndrome + vestibular syndrome + cerebellar ataxia. EDSS = 3

Patient 3	F	3	None	None	Dysarthria + gait disorder	Dysarthria + static and kinetic cerebellar syndrome. EDSS = 3

Patient 4	M	10	None	None	Seizures + altered consciousness + gait disorder + decreased vision + vomiting	Dysarthria + dystonia + cerebellar syndrome + proprioceptive dysfunction + nystagmus + 7th nerve paralysis + decreased vision. EDSS = 2

Patient 5	F	17	None	Type 1 diabetes	Decreased vision + sphincter dysfunction + weakness and numbness of lower limbs	Paraparesis + pyramidal syndrome + cerebellar syndrome + proprioceptive dysfunction. EDSS = 4.5

Patient 6	M	10	None	Type 1 diabetes	Right hemibody weakness + paresthesia	Right hemiplegia. EDSS = 5

Patient 7	F	17	Bipolar disorder	None	Left hemibody weakness + paresthesia	Left hemiparesis + tactile and pain sensory dysfunction. EDSS = 3.5

Patient 8	M	4	None	Asthma	Headache + left hemibody weakness + hand movement disorder + vomiting	Left hemiparesis + central facial paralysis + hand dystonia. EDSS = 3.5

Patient 9	F	16	None	None	Left upper limb weakness + paresthesia	Left pyramidal syndrome + hemibody hypoesthesia + sensory level C4. EDSS = 2

Patient 10	F	14	None	None	Dizziness + cervical movement disorder + vomiting	Cervical dystonia + pyramidal syndrome + vestibular syndrome + paraparesis. EDSS = 4

Patient 11	M	12	None	None	Dizziness + vomiting + diplopia	Vestibular syndrome + pyramidal syndrome. EDSS = 1.5

Patient 12	F	15	None	None	Tremor of upper limbs + convergent strabismus + diplopia + urinary dysfunction	Dysarthria + right convergent strabismus + nystagmus + cerebellar syndrome. EDSS = 2

Patient 13	F	7	None	None	Gait disorder + hand movement disorder	Dystonia + myoclonus + tremor. EDSS = 3

Patient 14	F	17	None	Hypothyroidism	Lower limb weakness + hemibody paresthesia + facial paralysis + hand movement disorder	Spastic paraparesis + pyramidal syndrome + hemibody hypoesthesia. EDSS = 3

Patient 15	M	4.5	None	None	Hemibody weakness + facial paralysis	Right hemiparesis. EDSS = 2

Patient 16	F	12	None	None	Strabismus + facial paralysis + gait disorder + diplopia + dizziness + swallowing difficulties	Pseudobulbar syndrome + proprioceptive dysfunction. EDSS = 6

Patient 17	F	17	None	None	Four limbs weakness + paresthesia	Left lower limb paresis + thermic and pain sensory dysfunction + proprioceptive dysfunction. EDSS = 4.5

Patient 18	F	16	None	None	Paresthesia + tremor + facial paralysis + lower limb weakness	Right hemiparesis + tremor + kinetic cerebellar syndrome + pyramidal syndrome. EDSS = 2,5

Patient 19	F	4	None	None	Dysarthria + hemibody weakness	Spasmodic laughter and weeping + dysarthria + tetraparesis + cerebellar syndrome. EDSS = 4,5

Patient 20	F	8	None	None	Left hemibody weakness	Left hemiparesis. EDSS = 2

Patient 21	F	13	None	Type 1 diabetes	Paresthesia + hemibody weakness	Flaccid paraparesis + sensory level D3. EDSS = 4

EDSS: Expanded Disability Status Scale; F: female; M: male.

**Table 2:** CSF analysis and neuroimaging findings in our series.

	CSF analysis (at onset)	Repeat CSF analysis	Brain and spinal MRI	Neurophysiological studies
Patient 1	Positive OCB	—	Periventricular, subcortical hyperintense lesions on T2 and FLAIR weighted images with right thalamus and pontine involvement and gadolinium enhancement	Normal VEP

Patient 2	Positive OCB, increased protein level	—	Brainstem, cerebellar, and insular subcortical hyperintense lesions on T2 and FLAIR weighted images without gadolinium enhancement	Optic neuritis

Patient 3	Normal	Positive OCB	Periventricular, subcortical hyperintense lesions on T2 and FLAIR weighted images with brainstem and cerebellar involvement	Optic neuritis

Patient 4	Negative OCB, increased protein level	Positive OCB	Subcortical hyperintense lesions on T2 and FLAIR weighted images with basal ganglia, brainstem, and posterior cordonal involvement	Optic neuritis

Patient 5	Positive OCB, increased protein level	—	Periventricular and subcortical hyperintense lesions on T2 and FLAIR weighted images with internal capsule, corpus callosum, thalamic, brainstem, and cerebellar peduncle involvement, extensive posterior spinal abnormalities, and gadolinium enhancement (Figure 1)	Optic neuritis, altered BAEP and SEP

Patient 6	Normal	Positive OCB	Periventricular and subcortical hyperintense lesions on T2 and FLAIR weighted images with brainstem and cervical involvement without gadolinium enhancement	Altered BAEP

Patient 7	Negative OCB, increased protein level	Normal	Periventricular hyperintense lesions on T2 and FLAIR weighted images with “onion bulb” appearance of one lesion and gadolinium enhancement	Altered BAEP

Patient 8	Normal	Positive OCB	Periventricular and semiovale center hyperintense lesions on T2 and FLAIR weighted images with internal capsule involvement	Altered SEP

Patient 9	Negative OCB, increased protein level	Positive OCB	Subcortical hyperintense lesions on T2 and FLAIR weighted images with cervical involvement and gadolinium enhancement	Altered SEP

Patient 10	Positive OCB	—	Periventricular, subcortical, semiovale center hyperintense lesions on T2 and FLAIR weighted images with corpus callosum, peduncular, pontine, and cerebellar involvement and cervical and dorsal abnormalities	Not performed

Patient 11	Increased cell count	Normal	Periventricular, subcortical, semiovale center hyperintense lesions on T2 and FLAIR weighted images with corpus callosum and cerebellar peduncles involvement and gadolinium enhancement	Normal VEP

Patient 12	Normal	Positive OCB	Corpus callosum hyperintense lesions on T2 and FLAIR weighted images with cerebellar and brainstem involvement and cervical abnormalities with gadolinium enhancement	Optic neuritis

Patient 13	Increased cell count	Normal	Periventricular hyperintense lesions on T2 and FLAIR weighted images with cervical involvement	Optic neuritis

Patient 14	Positive OCB, increased protein level and cell count	Positive OCB with normal cell count	Periventricular hyperintense lesions on T2 and FLAIR weighted images with cervical involvement and gadolinium enhancement	Optic neuritis, altered SEP

Patient 15	Increased cell count	Positive OCB with normal cell count	Periventricular hyperintense lesions on T2 and FLAIR weighted images	Normal VEP

Patient 16	Normal	Positive OCB	Periventricular hyperintense lesions on T2 and FLAIR weighted images with cerebellar and brainstem involvement	Not performed

Patient 17	Normal	Positive OCB	Periventricular hyperintense lesions on T2 and FLAIR weighted images with cervical involvement	Optic neuritis, altered SEP

Patient 18	Positive OCB	—	Periventricular and semiovale center hyperintense lesions on T2 and FLAIR weighted images with corpus callosum, brainstem, and cerebellar involvement	Optic neuritis, altered SEP

Patient 19	Positive OCB, increased protein level and cell count	Positive OCB with normal cell count	Periventricular hyperintense lesions on T2 and FLAIR weighted images with brainstem involvement	Bilateral optic neuritis

Patient 20	Not performed	—	Periventricular hyperintense lesions on T2 and FLAIR weighted images with “onion bulb” aspect of some lesions	Optic neuritis, altered SEP

Patient 21	Positive OCB and increased protein level	—	Subcortical hyperintense lesions on T2 and FLAIR weighted images with cerebellar, cervical, and dorsal involvement	Altered SEP

OCB: oligoclonal bands; BAEP: brain auditory evoked potentials; SEP: sensory evoked potentials; VEP: visual evoked potentials.

**Table 3:** Therapeutic management and follow-up data in our series.

	Immunomodulatory treatment	MS form	Relapse rate before immunomodulatory treatment (per year)	Relapse rate after immunomodulatory treatment (per year) and EDSS score
Patient 1	Interferon *β* 1a	RRMS	2	0, EDSS = 1
Patient 2	Interferon *β* 1a	RRMS	1	1, EDSS = 1
Patient 3	Interferon *β* 1a	RRMS	2	0, EDSS = 1.5
Patient 4	Interferon *β* 1a	RRMS	1	0, EDSS = 1
Patient 5	Interferon *β* 1a	RRMS	5	1.33, EDSS = 1
Patient 6	Interferon *β* 1b	RRMS	1	0, EDSS = 0
Patient 7	Interferon *β* 1a	RRMS	0,5	0, EDSS = 1
Patient 8	Interferon *β* 1a	RRMS	3	0.3, EDSS = 1
Patient 9	Interferon *β* 1a	RRMS	3	0.6, EDSS = 1
Patient 10	None	RRMS	—	—, EDSS = 0.5
Patient 11	None	RRMS	—	—, EDSS = 0.5
Patient 12	Interferon *β* 1a	RRMS	2	0.33, EDSS = 2
Patient 13	None	RRMS	—	—, EDSS = 1
Patient 14	Interferon *β* 1b	RRMS	2	1, EDSS = 1
Patient 15	Interferon *β* 1a	RRMS	4	0.2, EDSS = 1
Patient 16	Interferon *β* 1a	RRMS	1	0.3, EDSS = 1.5
Patient 17	Interferon *β* 1b	RRMS	2	1, EDSS = 1
Patient 18	Interferon *β* 1a	RRMS	4	1, EDSS = 1.5
Patient 19	Interferon *β* 1a switched with interferon *β* 1b	RRMS then SPMS	0.25	0, EDSS = 3.5
Patient 20	None	RRMS	—	—, EDSS = 1
Patient 21	Interferon *β* 1a	RRMS	1	0.2, EDSS = 1

RRMS: relapsing-remitting multiple sclerosis; SPMS: secondary progressive multiple sclerosis.

## References

[1] Absoud M., Lim M. J., Chong W. K. (2013). Paediatric acquired demyelinating syndromes: Incidence, clinical and magnetic resonance imaging features. *Multiple Sclerosis Journal*.

[2] Krupp L. B., Tardieu M., Amato M. P. (2013). International Pediatric Multiple Sclerosis Study Group criteria for pediatric multiple sclerosis and immune-mediated central nervous system demyelinating disorders: revisions to the 2007 definitions. *Multiple Sclerosis Journal*.

[3] Waldman A., Ghezzi A., Bar-Or A., Mikaeloff Y., Tardieu M., Banwell B. (2014). Multiple sclerosis in children: An update on clinical diagnosis, therapeutic strategies, and research. *The Lancet Neurology*.

[4] Dell’Avvento S., Sotgiu M. A., Manca S., Sotgiu G., Sotgiu S. (2016). Epidemiology of multiple sclerosis in the pediatric population of Sardinia, Italy. *European Journal of Pediatrics*.

[5] Renoux C., Vukusic S., Confavreux C. (2008). The natural history of multiple sclerosis with childhood onset. *Clinical Neurology and Neurosurgery*.

[6] Tintoré M., Arrambide G. (2009). Early onset multiple sclerosis: The role of gender. *Journal of the Neurological Sciences*.

[7] Nielsen N. M., Westergaard T., Frisch M. (2006). Type 1 diabetes and multiple sclerosis: a Danish population-based cohort study. *JAMA Neurology*.

[8] Tettey P., Simpson S., Taylor B. V., Van Der Mei I. A. F. (2015). The co-occurrence of multiple sclerosis and type 1 diabetes: shared aetiologic features and clinical implication for MS aetiology. *Journal of the Neurological Sciences*.

[9] Mehanna R., Jankovic J. (2013). Movement disorders in multiple sclerosis and other demyelinating diseases. *Journal of the Neurological Sciences*.

[10] Sudhakar S. V., Muthusamy K., Mani S., Gibikote S., Shroff M. (2016). Imaging in Pediatric Demyelinating and Inflammatory Diseases of the Brain- Part 1. *The Indian Journal of Pediatrics*.

[11] Banwell B., Krupp L., Kennedy J. (2007). Clinical features and viral serologies in children with multiple sclerosis: a multinational observational study. *The Lancet Neurology*.

[12] Narula S., Banwell B. (2015). Treatment of multiple sclerosis in children and its challenges. *Presse médicale (Paris, France : 1983)*.

[13] Ghezzi A., Amato M. P., Makhani N., Shreiner T., Gärtner J., Tenembaum S. (2016). Pediatric multiple sclerosis: Conventional first-line treatment and general management. *Neurology*.

[14] Pena J. A., Lotze T. E. (2013). Pediatric multiple sclerosis: Current concepts and consensus definitions. *Autoimmune Diseases*.

