# Supplementary material for: Pediatric Multiple Sclerosis in Tunisia: A Retrospective Study over 11 Years
Source: Biomed Res Int. 2017 Nov 7;2017:4354826. doi: 10.1155/2017/4354826 (PMC5697413; doi:10.1155/2017/4354826)
Supplement: Supplementary file 1 — More detailed information about demographics of Tunisia and presentation of our center. [file 4354826.f1.doc]

# Supplementary material file

# 1. Demographics of Tunisia **(**National Institute of Statistics; Tunisia) :

-Average number of total population (2005-2016): 10618933+/-399773 ; 95% Confidence interval : [10618863- 10619002]

-Average number of children less than 15 years old (2005–2016): 2555541+/-55557; 95% Confidence interval : [2555522- 2555561]

- Average number of pediatric population aged between 15 and 18 years (2005-2016): 934791+/-90631; 95% Confidence interval : [934738- 934791]

-Average number of pediatric population aged under 18 years (2005-2016): 3490333+/-62128; 95% Confidence interval : [3490314-3490352]

# 2. Presentation of our center:

# **Department of Child and Adolescent Neurology at National Institute Mongi Ben Hmida of Neurology is a** tertiary care research center and it is **the major referral center for children with neurological disorder. It serves a population of over than three million with an average number of outpatient and emergency visits: 20 000 per year**
